# Novel Polymorphisms in *Plasmodium falciparum* ABC Transporter Genes Are Associated with Major ACT Antimalarial Drug Resistance

**DOI:** 10.1371/journal.pone.0020212

**Published:** 2011-05-25

**Authors:** Maria Isabel Veiga, Pedro Eduardo Ferreira, Louise Jörnhagen, Maja Malmberg, Aminatou Kone, Berit Aydin Schmidt, Max Petzold, Anders Björkman, Francois Nosten, Jose Pedro Gil

**Affiliations:** 1 Malaria Research Lab, Department of Medicine, Karolinska Institutet, Stockholm, Sweden; 2 Drug Resistance and Pharmacogenetics Group, Centre of Molecular and Structural Biomedicine, Institute of Biotechnology and Bioengineering, University of Algarve, Faro, Portugal; 3 Nordic School of Public Health, Gothenburg, Sweden; 4 Shoklo Malaria Research Unit, Mae Sot Tak, Thailand; 5 Faculty of Tropical Medicine, Mahidol University, Bangkok, Thailand; 6 Nuffield Department of Clinical Medicine, Centre for Clinical Vaccinology and Tropical Medicine, University of Oxford, Oxford, United Kingdom; 7 Division of Pharmacogenetics, Department of Physiology and Pharmacology, Karolinska Institutet, Stockholm, Sweden,; 8 Laboratory of Molecular Anthropology and Health, Department of Anthropology, Binghamton University, Binghamton, New York, United States of America; Kenya Medical Research Institute-Wellcome Trust Research Programme, Kenya

## Abstract

Chemotherapy is a critical component of malaria control. However, the most deadly malaria pathogen, *Plasmodium falciparum*, has repeatedly mounted resistance against a series of antimalarial drugs used in the last decades. Southeast Asia is an epicenter of emerging antimalarial drug resistance, including recent resistance to the artemisinins, the core component of all recommended antimalarial combination therapies. Alterations in the parasitic membrane proteins Pgh-1, PfCRT and PfMRP1 are believed to be major contributors to resistance through decreasing intracellular drug accumulation. The *pfcrt, pfmdr1* and *pfmrp1* genes were sequenced from a set of *P.falciparum* field isolates from the Thai-Myanmar border. *In vitro* drug susceptibility to artemisinin, dihydroartemisinin, mefloquine and lumefantrine were assessed. Positive correlations were seen between the *in vitro* susceptibility responses to artemisinin and dihydroartemisinin and the responses to the arylamino-alcohol quinolines lumefantrine and mefloquine. The previously unstudied *pfmdr1* F1226Y and *pfmrp1* F1390I SNPs were associated significantly with artemisinin, mefloquine and lumefantrine *in vitro* susceptibility. A variation in *pfmdr1* gene copy number was also associated with parasite drug susceptibility of artemisinin, mefloquine and lumefantrine. Our work unveils new candidate markers of *P. falciparum* multidrug resistance *in vitro*, while contributing to the understanding of subjacent genetic complexity, essential for future evidence-based drug policy decisions.

## Introduction

In the 21st century, malaria is still regarded as a major global infectious disease, with a high pediatric mortality toll in the Developing World. Chemotherapy is a central tool for management of the disease. Unfortunately, the *Plasmodium falciparum* parasite, the most deadly human malaria pathogen, has shown an extraordinary ability to develop resistance to all antimalarials employed. From the first reports involving quinine [1], to the ultimate demise of chloroquine in the late 20^th^ century, drug resistance has been a central factor in the expansion of the disease, associated with massive mortality and morbidity [2]. Now, in the 21st century, the introduction of new treatment strategies centered on the combinatorial use of short lived artemisinin derivatives with long half-life drugs (artemisinin combination therapy, ACT) has been able to halt resistance and disease spread for the first time in decades. After its spectacular decrease in incidence following ACT introduction in some regions [3], this has encouraged optimism for the prospects of eliminating malaria entirely [4]. Unfortunately, *P. falciparum* is again demonstrating its remarkable capacity to evolve mechanisms of avoiding drug action. Among the presently employed ACT drugs, decreased sensitivity has been reported to all the quinoline partner drugs in use, including amodiaquine [5]–[7], lumefantrine [8], [9] and mefloquine [10]. More recently, *P.falciparum* isolates in Cambodia were shown to have developed tolerance to artemisinin, the core component of the combinations [11], [12]. This tolerance is characterized by prolonged parasite clearance after treatment [12].

For most ACT partner drugs, the mechanisms behind antimalarial drug resistant phenotypes are known to be dependent on alterations in the food vacuole localized proteins Pgh-1 (*pfmdr1*) and PfCRT (*pfcrt*). Both PfCRT and Pgh-1 are drug transporters believed to contribute to *P. falciparum* drug resistance through the decrease of antimalarial accumulation in the target cellular compartment. PfCRT transports drugs from the food vacuole lumen to the cytoplasm [13], [14], an action particularly important to the development of resistance, considering that several antimalarials, such the 4-aminoquinolines amodiaquine, desethylamodiaquine and chloroquine, act in this organelle. In contrast, Pgh-1 has been proposed to actively pump drugs from the cytoplasm towards the food vacuole [15], which is protective against antimalarials operating on cytoplasmic targets. Such agents putatively include the aminoalcohol-quinolines (AAQs) [10], and possibly artemisinin and its derivatives [16].

There is an emerging idea that these resistance phenomena actually involve genetic contributions beyond the genes already mentioned [17], [18]. Accordingly, it is likely that another player could be added to the two transmembrane proteins discussed earlier: the parasite multidrug resistance protein homologue, PfMRP1 [19]–[21]. This class of ABC transporter is involved in drug evasion in a large range of organisms, and likely plays a similar function in *P. falciparum*. In support of this, *pfmrp1* has been proposed to participate in *in vivo* and/or *in vitro* parasite response to chloroquine [18], [22], quinine [18], [23], lumefantrine [17] and artemisinin [22].

A full account of the simultaneous participation of each of these genes in parasite drug susceptibility to multiple drugs has not yet been performed in detail, in part due to the diversity of their full open reading frame (ORF). In this report, we have characterized the phenotype and genotype of a set of *P. falciparum* adapted parasites from the area of Mae Sot, Western Thailand-a known epicenter for multidrug resistance. The work has unveiled different genetic backgrounds containing a combination of different polymorphisms associated with resistance to antimalarial drugs used in ACT.

## Results

### *In vitro* antimalarial drug susceptibility

**Figure 1:**
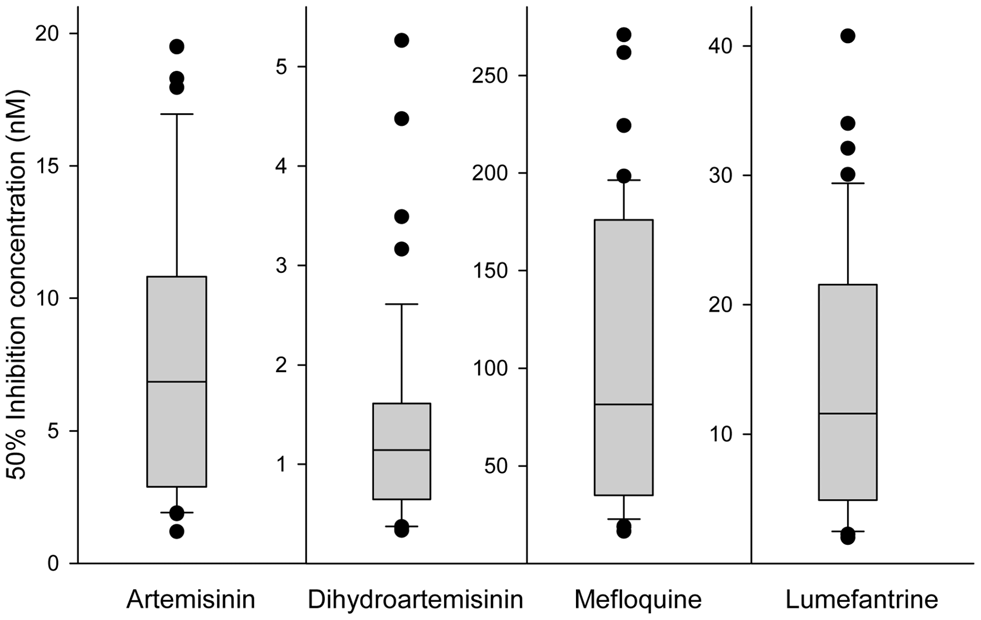
*In vitro* antimalarials 50% inhibition concentration variance within the isolates. Data are represented in boxplot. The lower and upper quartiles of the box are 25^th^ and 75^th^ percentile, respectively and the band near the middle of the box is the median. End of the whiskers represent the 10^th^ and 90^th^ percentile. Dots represent outliers.

50% and 90% inhibitory concentration (IC_50_ and IC_90_) values were measured for artemisinin (ART), dihydroartemisinin (DHA), mefloquine (MQ) and lumefantrine (LUM) in 46 parasite field isolates originating from the Thai-Myanmar border. A relatively large range in IC values was observed for most of the antimalarials tested, including the artemisinin-type drugs. For these, the median IC_50_ values (Max-Min) were 7.4 nM (1.2–19.5 nM) for ART and 1.2 nM (0.3–5.3 nM) for DHA. For the arylaminoalcohols, IC_50_ values were: MQ and LUM, 92.5 nM (16.5–270.8 nM) and 11.9 nM (2.0–40.8 nM), respectively (Figure 1 and isolates individual data in Table S1).

There is a significant correlation between the parasitic response to structurally associated drugs such as sesquiterpene lactone (ART and DHA) and aminoalcohol quinolines (MQ and LUM) (Table 1). Interestingly, even though not structurally similar, the amino alcohol quinolines group and the artemisinin-type compounds form a cluster of cross response (P<0.001).

**Table 1:** Correlations of *in vitro* IC's values between the different drugs tested in 46 field isolates as well as associations between the IC's values and the major polymorphisms in *pfmdr1* and *pfmrp1* genes.

	*In vitro* drug IC's significance§							*pfmdr1* polymorphisms significance¤						*pfmrp1* polymorphisms significance¤					
	ART IC_90_	DHA IC_50_	DHA IC_90_	MQ IC_50_	MQ IC_90_	LUM IC_50_	LUM IC_90_	130 E-K	184 Y-F	249 syn	750 A-T	1226 F-Y	CNV	325 N-S	572 F-L	785 H-N	876 I-V	1007 T-M	1390 F-I
**ART IC_50_**	**<0.001** (0.97)	**<0.001** (0.68)	**<0.001** (0.66)	**<0.001** (0.82)	**<0.001** (0.69)	**<0.001** (0.88)	**<0.001** (0.80)	0.855	0.155	0.855	0.528	**0.003**	**<0.001**	0.628	0.141	0.181	0.791	0.181	**0.007**
**ART IC_90_**		**<0.001** (0.6)	**<0.001** (0.63)	**<0.001** (0.82)	**<0.001** (0.73)	**<0.001** (0.81)	**<0.001** (0.77)	0.942	0.353	0.942	0.626	**0.005**	**<0.001**	0.533	0.120	0.145	0.623	0.145	**0.004**
**DHA IC_50_**			**<0.001** (0.90)	**0.001** (0.49)	**0.027** (0.33)	**<0.001** (0.70)	**<0.001** (0.54)	0.395	0.467	0.395	0.596	0.354	0.284	0.925	0.744	0.416	0.926	0.416	0.163
**DHA IC_90_**				**0.004** (0.41)	**0.075** (0.27)	**<0.001** (0.63)	**<0.001** (0.51)	0.974	0.538	0.974	0.870	0.292	0.152	0.925	0.566	0.398	0.922	0.398	0.192
**MQ IC_50_**					**<0.001** (0.96)	**<0.001** (0.81)	**<0.001** (0.80)	0.606	0.249	0.606	0.342	**0.004**	**<0.001**	0.835	0.116	0.174	0.722	0.174	**0.003**
**MQ IC_90_**						**<0.001** (0.66)	**<0.001** (0.72)	0.458	0.389	0.458	0.233	**0.004**	**<0.001**	0.886	0.063	0.113	0.976	0.113	**0.004**
**LUM IC_50_**							**<0.001** (0.90)	0.527	0.163	0.527	0.320	**0.004**	**<0.001**	0.755	0.238	0.209	0.801	0.209	**0.015**
**LUM IC_90_**								0.181	0.249	0.181	0.087	**0.001**	**<0.001**	0.526	0.124	0.224	0.903	0.224	**0.022**

§ Cells show P-value (2-tailed) and in brackets the Pearson correlation coefficient between the IC's values of the different antimalarial tested in the isolates. P value<0.05 is considered significant (2-tailed).

¤ Cells show P-value (2-tailed) of the linear association analysis between the main polymorphisms found in the isolates (Table 2) and the *in vitro* drug IC's values. Isolates that harbors both alleles (mixed infection) in the determined polymorphic region were excluded from the statistical analysis. T-test with equal variances assumed was used to test differences. Bold numbers are considered significant for T-test (P<0.05). For comparison, Bonferroni-adjusted levels of significance would be: P value<0.0042.

ART: artemisinin, DHA: dihydroartemisinin, MQ: mefloquine, LUM: lumefantrine, IC_50_ and IC_90_: Inhibition Concentration of 50% and 90%. CNV: copy number variation.

### Polymorphisms detected in *pfmdr1*, *pfmrp1* and *pfcrt*

Sequence analysis of the full *pfmdr1* open reading frame (ORF) within the isolates revealed 7 SNPs, including two well studied polymorphic amino acid positions (Y184F and N1042D), as well as 4 other non-synonymous at positions E130K, A750T, S784L, F1226Y, recently described in parasites from Western Cambodia [24], and one synonymous at genomic position a747g (Table 2). Three variants were found within the triplet nucleotide AAT microsatellite hinge located near the central region of the gene (Table 2) where 31/46 of the isolates carry an insertion of one more AAT relative to 3D7 clone reference. Sequencing chromatograms of this gene revealed one isolate with mixed infection present at amino acid position 130, 249 and 750.

**Table 2:** Single Nucleotide Polymorphisms in the studied transporter genes.

	Nucleotide position	Triplet nucleotide change	Amino acid position	Amino acid change	Frequency (proportion)
*pfmdr1*					
	388	GAA-AAA	130	E-K	0.13 (6/46)+1mix
	551	TAT-TTT	184	Y-F	0.09 (4/46)
	747	GGA-GGG	249	syn	0.13 (6/46)+1mix
	1972–1980	del 3xAAT	658	_ _ _	0.09 (4/46)
	1978–1980	del AAT	660	_	0.24 (11/46)
	1980	ins AAT	660+1	+N	0.67 (31/46)
	2248	GCA-ACA	750	A-T	0.15 (7/46)+1mix
	2351	TCA-TTA	784	S-L	0.02 (1/46)
	3124	AAT-GAT	1042	N-D	0.02 (1/46)
	3677	TTT-TAT	1226	F-Y	0.50 (23/46)+1mix
*pfmrp1*					
	300	GCA-GCT	100	syn	0.04 (2/46)
	571	CAT-TAT	191	H-Y	0.96 (44/46)
	974	AAT-AGT	325	N-S	0.20 (9/46)+1mix
	1309	TCA-GCA	437	S-A	0.96 (44/46)
	1716	TTC-TTG	572	F-L	0.39 (18/46)+1mix
	2353	CAT-AAT	785	H-N	0.50 (23/46)+1mix
	2626	ATA-GTA	876	I-V	0.74 (34/46)+1mix
	3020	ACG-ATG	1007	T-M	0.50 (23/46)+1mix
	3603	GAA-GAG	1201	syn	0.04 (2/46)
	4168	TTT-ATT	1390	F-I	0.17 (8/46)+2mix
	4451	GAA-GGA	1484	E-G	0.02 (1/46)
*pfcrt*					
	222	ATG-ATT	74	M-I	1 (46/46)
	223	AAT-GAT	75	N-D	1 (46/46)
	225	AAT-GAA	75	N-E	1 (46/46)
	227	AAA-ACA	76	K-T	1 (46/46)
	658	GCC-TCC	220	A-S	1 (46/46)
	811	CAA-GAA	271	Q-E	1 (46/46)
	977	AAC-AGC	326	N-S	1 (46/46)
	1067	ATA-ACA	356	I-T	1 (46/46)
	1112	AGA-ATA	371	R-I	1 (46/46)

All Thai isolates carry an asparagine residue at position 86 and the SNP with the highest frequency detected was the F1226Y with 23/46 (50%) isolates being 1226Y. The remaining SNPs were found with low frequency (Table 2). Twenty six of the 46 isolates analyzed carried increased *pfmdr1* copy number, as detected by realtime PCR. Specifically, 18 carried two copies, while 8 carried more than three copies (Table S1).

Analysis of the sequencing chromatograms of the *pfmrp1* gene revealed one isolate with mixed infection present at a.a. position 325, 572, 785, 876 and 1007. This isolate does not correspond to the same mixed isolate detected with *pfmdr1* gene sequencing. In total 11 SNPs (of which 2 synonymous) were identified in the *pfmrp1* ORF. All of these SNPs were published following our previous global survey of the biodiversity of this gene [17]. SNPs at positions 191, 325, 437, 572, 785, 876, 1007, 1390 were found in the isolates with elevated occurrence (Table 2). Increased *pfmrp1* gene copy number was not detected.

The *pfcrt* full open reading frame of each isolate sequenced from cDNA revealed that all isolates (46/46) carried the *pfcrt* Dd2-like open reading frame (ORF) haplotype (C72 /74I/75E/76T/220S/271E/326S/356T/371I) (Table 2).

After the *in vitro* analysis in the laboratory for drug susceptibility, the DNA was extracted from all isolates again to check for variance and/or selection. The recheck was solely performed in the polymorphisms with significant association with the *in vitro* phenotype. These were the *pfmdr1* CNV, as well as the *pfmdr1* F1226Y and *pfmrp1* F1390I SNPs. Comparison of the DNA extracted before and after the parasites *in vitro* adaptation and expansion, revealed that two isolates lost copies in *pfmdr1* gene during the procedure, changing from 2 to 1 copy (Table S1). Out of the 46 isolates analyzed, 24 carried increased *pfmdr1* copy number, specifically, 16 carried two copies, while 8 carried more than three copies. Pyrosequencing of position F1226Y in *pfmdr1* and F1390I in *pfmrp1* performed in the DNA extracted after the drug susceptibility tests, revealed isolates with mixed infections (both alleles) at these polymorphic regions. Table S1 specifies the characteristics of each isolate including the individual genotype before and after the drug susceptibility tests performed.

Table 2 shows the genotype frequency of these three most relevant polymorphisms, from the re-extracted DNA, as this would reflect the genotype at that point in time. Table 1 also shows statistical values using data from the re-extracted DNA analysis for these three polymorphisms as this will reflect a more valid association with the *in vitro* drug susceptibility tests performed. To note that this genotype variance before and after adaptation, did not change the determined statistically significant genotype-phenotype association.

### *In vitro* phenotype-genotype associations

**Figure 2:**
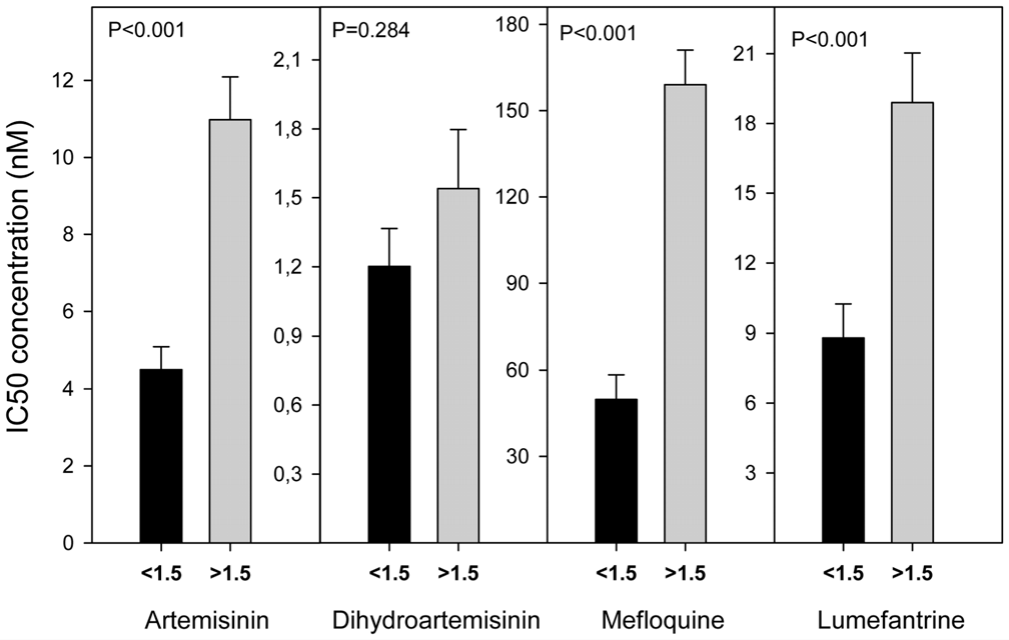
Significant association of *pfmdr1* copy number variation and in vitro IC_50_'s for ART, MQ and LUM. Significant relationship between average *in vitro* drugs tested IC_50_'s and *pfmdr1* CNV polymorphism. Data described in Table S2. <1.5: 1 gene copy of *pfmdr1* (#22 isolates). >1.5: 2 and 3 gene copies of *pfmdr1* (#24 isolates). T-test statistics was applied. P value <0.0042 is consider significant after Bonferroni correction. Error bars represent SE.

**Figure 3:**
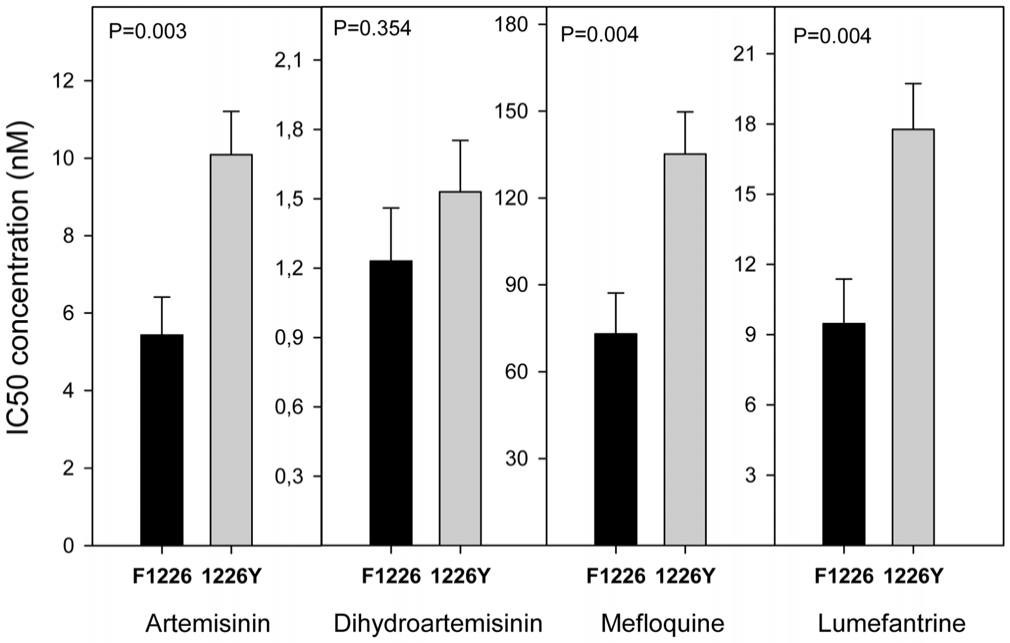
Significant association of *pfmdr1* F1226Y and IC_50_'s for ART, MQ and LUM. Significant relationship between average *in vitro* drugs tested IC_50_'s and *pfmdr1* F1226Y polymorphism. Data described in Table S2. Black bars are average IC_50_ of 22 isolates with F1226 allele. Grey bars are average IC_50_ of 23 isolates with 1226Y allele. T-test statistics was applied. P value <0.0042 is consider significant after Bonferroni correction. Error bars represent SE.

**Figure 4:**
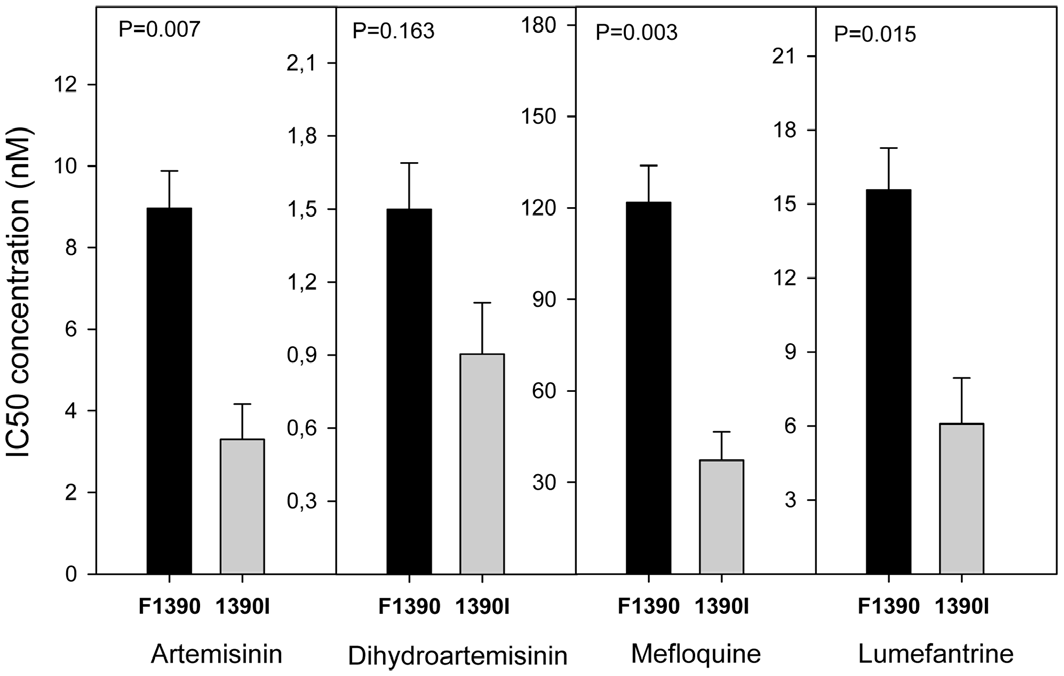
Significant association of *pfmrp1* F1390I and IC_50_'s for ART, MQ and LUM. Significant relationship between average *in vitro* drugs tested IC_50_'s and *pfmrp1* F1390I polymorphism. Data described in Table S2. Black bars are average IC_50_ of 36 isolates with F1390 allele. Grey bars are average IC_50_ of 8 isolates with 1390I allele. T-test statistics was applied. P value <0.0042 is consider significant after Bonferroni correction. Error bars represent SE.

Since all isolates had the same haplotype for the *pfcrt* gene, a possible association could not be assessed between the *pfcrt* SNPs and IC's values. As previously observed [8], [10], [16], [25], and herein confirmed, an increased gene copy number of *pfmdr1* was clearly associated with higher IC_50_'s for MQ, LUM and ART (P<0.0042) (Table 1, Figure 2). From the seven SNPs found in *pfmdr1,* only one, F1226Y, was found to be associated with decreased sensitivity to ART, MQ and LUM IC_50_ and IC_90_ (Table 1, Figure 3). The average IC_50_ (±SE) of the *pfmdr1* 1226Y allele was 10.09(±1.12), 135.18(±14.57), and 17.77(±1.95) nM, values significantly higher compared with the non-carriers: 5.43(±0.98), 73.06(±14.08) and 9.47(±1.90) nM, for each drug, respectively. Among the 11 SNPs found in *pfmrp1* (Table 2), F1390I was documented to significantly modulate the *in vitro* drug response phenotypes (Table 1). Carriers of the F1390 allele were less susceptible to ART, MQ and LUM, with average IC_50_'s of 8.96(±0.92), 121.71(±12.17) and 15.56(±1.71) nM, compared to 1390I carriers with values of 3.30(±0.86), 37.25 (±9.31) and 6.08(±1.87) nM, respectively (Figure 4). Interestingly, even though *pfmdr1* F1226Y and *pfmrp1* F1390I alleles have very similar association profiles, both seem to represent independent actions, as they were not found together in the same Thai isolate in more instances than would randomly be expected (P>0.05).

**Figure 5:**
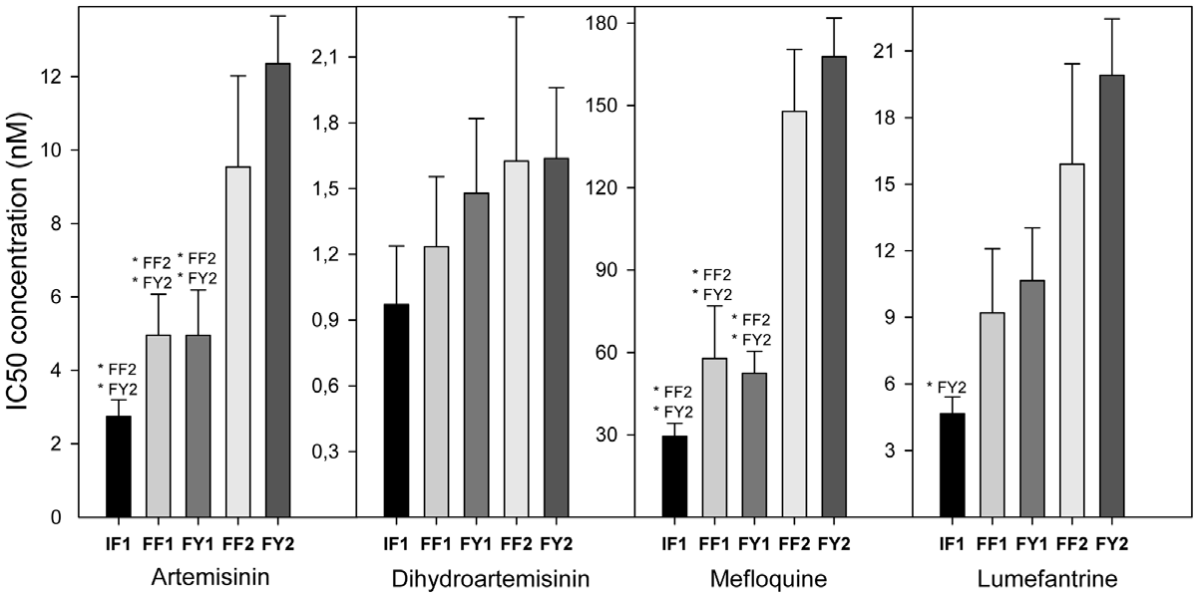
Haplotype and *in vitro* IC_50_'s association. Relationship between average *in vitro* drug tested IC_50_'s and *pfmrp1* F1390I, *pfmdr1* F1226Y and *pfmdr1* CNV polymorphisms. Bars represent different haplotypes. IF1 (#6 isolates): *pfmrp1* 1390I, *pfmdr1* F1226 and 1 gene copy of *pfmdr1*. FF1 (#9 isolates): *pfmrp1* F1390, *pfmdr1* F1226 and 1 gene copy of *pfmdr1*. FY1 (#5 isolates): *pfmrp1* F1390, *pfmdr1* 1226Y and 1 gene copy of *pfmdr1*. FF2 (#6 isolates): *pfmrp1* F1390, *pfmdr1* F1226 and >1 gene copy of *pfmdr1*. FY2 (#15 isolates): *pfmrp1* F1390, *pfmdr1* 1226Y and >1 gene copy of *pfmdr1*. T-test statistics was applied to see significant difference between haplotypes. *: P<0.05. Error bars represent SE.

We have further analyzed the effects of the available combinations (haplotypes) of the most relevant polymorphisms, i.e. the *pfmrp1* F1390I and *pfmdr1* F1226Y SNPs, as well as *pfmdr1* gene copy number variation (CNV). In particular, a trend for an identical progressive increase in IC_50_ was evident for the antimalarials ART, MQ and LUM (Figure 5), associated with the following sequence of haplotypes (from more sensitive to more **resistant** associated):

- IF1: *pfmrp1* 1390I+*pfmdr1* F1226+*pfmdr1* CNV<1.5;
- **F**F1: *pfmrp1* F1390+*pfmdr1* F1226+*pfmdr1* CNV<1.5;
- **FY**1: *pfmrp1* F1390+*pfmdr1* 1226Y+*pfmdr1* CNV<1.5;
- **F**F**2:** *pfmrp1* F1390+*pfmdr1* F1226+*pfmdr1* CNV>1.5;
- **FY2:** *pfmrp1* F1390+*pfmdr1* 1226Y+*pfmdr1* CNV>1.5.

Crystal structures of ABC transporters from *P. falciparum* are still not available. In lieu of this, an approach based on computational molecular modeling with known reference structures was applied to explore putative functional implications of relevant polymorphisms.

**Figure 6:**
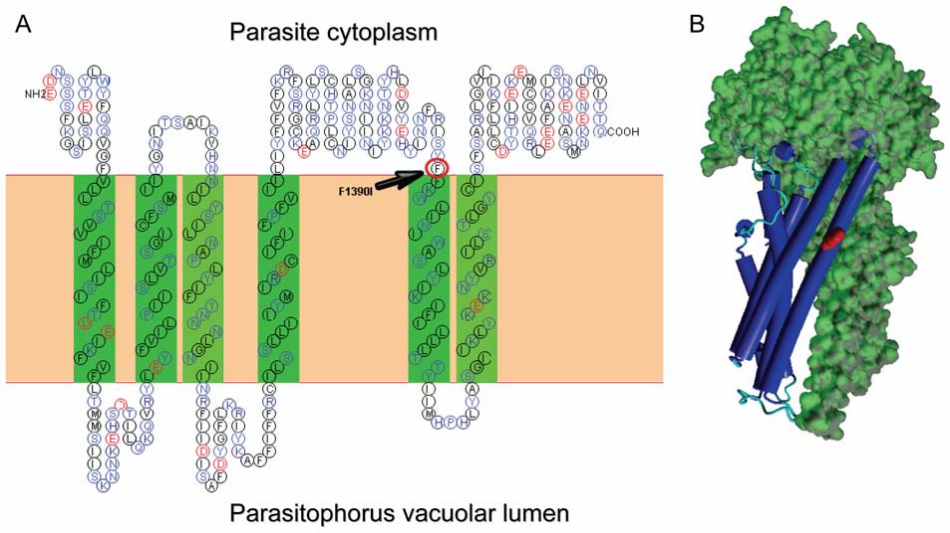
2D structure of PfMRP1 and F1390I SNP location. A: Secondary structure analysis of transmembrane spanning domain 2 (TSD2) of PfMRP1 and F1390I SNP localization. B: Computational homology modelling was performed at HHpred. Alignment for best template was performed using Psi-blast and with local alignment. The template from the best E-score alignment was based in the Msba ABC transporter crystal 3B60 then used in Modeller software to generate a model. Models were visualized with Yasara software.

As we previously reported [17], PfMRP1 contains 12 transmembranes domains (TMs) and 2 NBDs, presenting a full ABC transporter structure (i.e. 6+6 TMs), similar to Pgh-1 [26]. Residue 1390 is expected to be localized in transmembrane 11 (Figure 6A). Based on the Msba ABC transporter crystal 3B60 and using HHpred, a homology detection and structure prediction server, we were able to develop a model for the second transmembrane spanning domain 2 (Figure 6B).

The residues 1034 and 1042 of Pgh-1 are located in TM11, an important domain for antimalarial resistance [27]. This is likely due to alterations in a drug pocket region of the transporter. Similarly, the involvement of polymorphisms related to antimalarial resistance in TM11 of PfMRP1 suggests the existence of a drug-binding pocket in this region. Amino acid residue 1226 in Pgh-1 is localized in a non-conserved insertion in the reference structure, not allowing the possibility to perform homology modeling.

## Discussion

The global implementation of ACT has led to a significant world-wide reduction in malaria incidence. This, in turn, has motivated policymakers and donors to adopt a more optimistic view of the prospect of eliminating malaria completely in vast tropical endemic regions [4]. In order to make this a reality, and considering the epidemiological consequences of chloroquine resistance [2], we must understand the mechanisms behind incidences of parasite resistance to ACT. This is particularly urgent, as there is accumulating evidence of development of resistance in the field - or at least significant decreases in parasite response against artemisinin derivatives [11], [12] and to the long half-life ACT partner drugs [6], [9], [10], [16], [28], [29].

Through the search for drug resistance molecular markers, as expressed by their modulation of the specific parasite antimalarial *in vitro* IC's, our results reinforced the importance of *pfmdr1* copy number as a central factor in the response of *P. falciparum* to drugs of different structures. The association between increased copy number and decreased sensitivity to ART, MQ and LUM confirms previous associative data *in vivo* [8], [10], as do strictly controlled gene knock-down experiments in parasite clones [30].

Most relevant, we have identified SNPs that constitute new drug resistance candidate markers. These are comprised of two recently described polymorphisms: *pfmdr1* F1226Y [24] and *pfmrp1* F1390I [17]. Structural analysis performed for polymorphism F1390I in PfMRP1 showed it to be localized within transmembrane 11, which has been proposed to be part of a substrate pocket in several ABC transporters, including Pgh-1. The similar localization and involvement of this polymorphism for antimalarial resistance suggests it has an analogous roll to the S1034C and N1042D SNPs observed in Pgh-1 [27], [31]. Importantly, directed mutagenesis studies performed in the human and murine PfMRP1 have pointed to TM16, putatively equivalent to the PfMRP1 TM11 in functional terms, as a central component that defines transport specificity [32]–[36]. In this context, it is conceivable that the TM11 located F1390I SNP is changing the specificity of the interactions between the PfMRP1 and the antimalarials ART, MQ and LUM .

The *pfmdr1* F1226Y and *pfmrp1* F1390I SNPs seem to have an inter-independent, but very similar quantitative effect on the simultaneous response *in vitro* drug susceptibility of the parasite against the ART/MQ/LUM, which to a certain extent parallels what is observed with the *pfmdr1* increased copy number. In particular, successive accumulation of these SNPs, combined with the presence of *pfmdr1* CNV polymorphism, seem to have a cumulative contribution (Figure 5). As for the *pfmdr1* F1226Y SNP, it is conceivable that, as previously proposed for the *pfmdr1* 86N allele [37], its presence might enhance the contribution of the increased copy number of this gene. The preferential presence of F1226Y among the analyzed isolates with *pfmdr1* copy number amplifications might hence be the result of a drug driven selection, reinforcing the notion that the phenotypical consequences of the *pfmdr1* CNV polymorphism is modulated by the nature of the ORF sequence involved.

In this work we unveil a potential new candidate pathway towards resistance, particularly important for two of the most important ACTs used worldwide: artemether-LUM (Coartem^®^, Novartis, Basel) and artesunate-MQ (Artequin Paediatric^®^, Mepha Ltd, Aesch, Switzerland). It would be of interest to retrospectively explore *in vivo* trials with these combinations in search of confirmatory selection events for these mutations, in particular the case of artesunate-MQ, the definition of two categories of *pfmdr1* CNV. For the F1226Y SNP, its association with clinical failure might be of relevance. Interpreting the *pfmrp1* F1390I/ *pfmdr1* F1226Y/ *pfmdr1* CNV haplotype analysis, two observations deserve mention as is evident from figure 5. The introduction of the *pfmdr1* CNV is associated with an abrupt leap in MQ IC_50_ values, confirming the well established status of this type of mutation as a valuable specific marker for *P. falciparum* resistance against MQ [10], [16], [38]. The *pfmdr1* CNV alone seems to have a less dramatic but significant effect against ART and LUM, again confirming previous findings [8], [10], [27]. Fortunately, ART is not commonly used in ACT; the partners of LUM and MQ being artemether and artesunate, respectively.

Although previous reports have shown association of *pfmdr1* CNV with artesunate and DHA [8], [10], this study did not find any significant association of DHA with *pfmdr1* CNV neither with *pfmrp1* F1390I or *pfmdr1* F1226Y. Nevertheless, it showed significant positive correlation with ART, MQ and LUM *in vitro* susceptibility responses (Table 1).

Taken together, these data point towards the necessity for further research defining molecular markers for the development of resistance against the artemisinin derivative+amino-alcohol quinoline partner drugs, as these two structurally different classes of antimalarials seem to share, at least to some extent, mechanisms of drug action evasion. The *pfmrp1* F1390I and *pfmdr1* F1226Y SNPs, in conjugation with the well established *pfmdr1* CNV, represent likely candidates. The selection of these SNPs might be a pivotal step towards understanding Coartem^®^ resistance in locations where this ACT is a first line strategy, namely in a significant number of African countries, where *pfmdr1* CNV are presently rare, but nevertheless existent [39]–[41].

**Figure 7:**
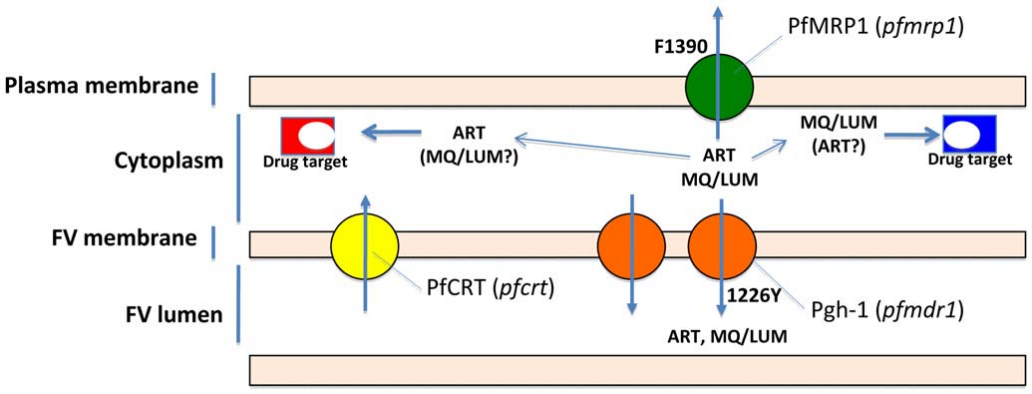
Proposed model for mode of action of the polymorphisms found. The central assumption is that ART, LUM and MQ drugs have their main pharmacological targets located in the cytoplasmic compartment while PfMRP1, operates by reducing the intra-cytoplasmatic concentrations by effluxing them out of the cell. Pgh-1, on its turn will contribute to further drug expulsion from the cytoplasm by transporting these drugs towards the lumen of the food vacuole.

The precise mechanistic contribution of each one of these mutations remains an open question. From our results and others, a simple model compiling the available data can be proposed (figure 7), partly extending from previous suggestions [17], [22], [29]. The central assumption is that ART, LUM and MQ drugs have their main pharmacological targets located in the cytoplasmic compartment [29] while PfMRP1, located in the plasma membrane, pumps them out of the cell, thereby reducing the intra-cytoplasmatic concentrations of the drugs. Pgh-1, inserted in the food vacuole membrane, would contribute to further drug expulsion from the cytoplasm by transporting these drugs towards the lumen of this organelle.

From our research, we are aware that the *pfmrp1* F1390I SNP is present in the African continent [17]. This is at least the case on the West coast where, incidentally, MQ has been traditionally considered to have lower efficacy compared with the Eastern regions of the continent. No information exists out of SE Asia concerning the *pfmdr1* F1226Y SNP, highlighting the need for more comprehensive studies on this SNP, particularly in Africa, where Coartem^®^ is the most common first line malaria treatment.

In conclusion, we propose novel potential markers of *in vitro* multidrug resistance against ACT drugs, particularly involving the pivotal artesunate-MQ and artemether-LUM combinations. These results further reinforce the complexity of emerging resistance to ACTs, consisting of several different molecular contributions, and hence multiple genetic markers.

## Materials and Methods

### Ethics statement

Collection protocols were approved by the Ethical Committee of the Faculty of Tropical Medicine, Mahidol University, Bangkok and Oxford Tropical Research Ethics Committee (OXTREC) at University of Oxford, UK.

The attending physician assured provision of written informed consent in the local language. After having obtained the patients or parents informed consent, venous blood was collected.

### Parasites

Isolates of *P. falciparum* were collected from malaria patients before treatment between the year 2002 and 2008 in Mae Sot, Thailand. Forty six isolates were chosen to be enrolled in this study. Parasites were cryo-preserved in liquid nitrogen until further use.

Culture adaptation of the isolates was performed in 5% human O^+^ RBCs in RPMI 1640 culture medium supplemented with 10% L-glutamine, 0.05% gentamicine (Gibco^®^/Invitrogen™, Carlsbad, CA, USA) and 10% human AB^+^ serum. The cultures were incubated at 37°C with addition of a gas mixture (5% O2, 5% CO2 in N2) and shook at 50 rpm.

### *In vitro* drug susceptibility assays

Chemosensitivity testing *in vitro* was performed with 4 antimalarial drugs. Artemisinin-282.33 MW, dihydroartemisinin-284.35 MW and mefloquine hydrochloride-414.77 MW, were purchased from Sigma-Aldrich (St louis, MO, USA) and lumefantrine-530 MW (LUM) from Novartis (Basel, Switzerland). The drugs were reconstituted in different solvents depending on their solubility. The IC's values were assessed using a Histidine-Rich Protein 2 based Double-Site Sandwich Enzyme-Linked Immunosorbent Assay [42]. Briefly, the parasite isolates were cultured (synchronized at ring stage containing 0,05% parasitaemia and 1,5% hematocrite) in the presence of serial dilutions of antimalarial drugs (ART, DHA, LUM and MQ). After 72 hours of culturing at 37°C in candle jars, cells were then lysed by freeze-thawing for ELISA analysis. Four independent assays were performed for each field isolate and 3D7 reference strain IC_50_ measured once a month for drug quality control. 3D7 strain was obtained from Prof. D. Walliker (Department of Animal and population genetics, University of Edinburgh, UK).

### Nucleic acids extraction

Blood samples from each culture-adapted isolate were centrifuged and 50 µL packed parasitized RBC frozen for DNA extraction. For RNA extraction 150 µL of PBS plus 300 µL of Lysis buffer (Applied Biosystems™, Fresno, CA, USA) were added to 50 µL packed parasitized RBC. The mix was kept at −20°C until RNA extraction. DNA and RNA extraction was carried out using an ABIPRISM^®^6100 Nucleic Acid PrepStation^®^ (Applied Biosystems™, Fresno, CA, USA) according to the recommendations of the manufacturer. cDNA synthesis was carried out using a High-Capacity cDNA Reverse Transcription Kit (Applied Biosystems, Fresno, CA, USA).

### Drug transporter genes open reading frame sequencing

All 46 isolates were fully sequenced for *pfmdr1* and *pfmrp1* genes from gDNA and the *pfcrt* gene was sequenced from cDNA. Amplification and sequencing primers for the *pfmrp1* were as described [17] and for *pfmdr1* and *pfcrt* genes the primers are shown in supplementary supporting information (Material and Methods S1). Amplicon fragments were sequenced by Macrogen Inc. (Seoul, Korea).

The Sequencher™ software versions 4.6 (Gene Codes Corporation, Ann Arbor, MI) was used to analyze the sequence output with the 3D7 sequence reference of PFE1150w (*pfmdr1*), PFA0590w (*pfmrp1*) and MAL7P1.27 (*pfcrt*) downloaded from Plasmo DataBase (http://plasmodb.org/).

### Gene copy number amplification of *pfmdr1* and *pfmrp1*

Relative quantification of *pfmdr1* and *pfmrp1* genes in all isolates was performed using housekeeping gene tubulin beta chain putative (PF10_0084). TaqMan^®^ probes and primers for *pfmdr1* were as described [10]. For *pfmrp1*, new primers (fw 5′-AGT AGA AGG AAG AGA CAT TCG AAC ATA-3′ and rev 5′-CAA AAG AAG ATT GAG CTA AAA TAC CAA-3′) and probe (6-FAM, MGBNFQ 5′-AAT AGA AAA GGA GAA GAT AG-3′) were designed (Applied Biosystems™, Fresno, CA, USA) to be performed as a multiplex using the same housekeeping gene primers and probe employed in *pfmdr1* analysis.

*P.falciparum* DNA from 3D7, K1 and FCB reference strains were used as calibrators and positive controls (known copy number variation for *pfmdr1* gene). Multiplex amplification reactions were done in triplicate in 96 well plates with 20 µl containing TaqMan^®^ Gene Expression Mastermix (Applied Biosystems), 300 nM of each forward and reverse primer, 100 nM of TaqMan^®^ probe from both target and housekeeping gene and 4 µL of gDNA. The thermal cycle profile was 50°C 2 min, 95°C 10 min and forty-five cycles of 95°C 15 s and 60°C 1 min. PCR was performed in ABI PRISM® 7000 Sequence Detection System (Applied Biosystems™, Fresno, CA, USA). The detection threshold was set above the mean baseline value for the first 6–15 cycles.

### Genotype re-analysis after culture adaptation

To check the existence of any variance or selection of parasites genotypes in the field isolates after *in vitro* adaptation, the DNA was extracted from the culture plates (row “A”/no drug control) in which the last tests for ICs was performed.

Genotyping was done only in the polymorphisms herein observed to be significantly associated with *in vitro* susceptibility: the *pfmdr1* CNV, *pfmdr1* F1226Y and *pfmrp1* F1390I. The method to recheck *pfmdr1* CNV was the same as described above. For *pfmdr1* F1226Y and *pfmrp1* F1390I pyrosequencing was the preferred method due to time/cost efficiency. The pyrosequencing protocol and primers were designed with Pyrosequencing assay design software, version 1.0 (Biotage AB, Uppsala, Sweden). Amplification conditions, pyrosequencing primers and the nucleotide dispensation orders are described in supplementary supporting information (Material and Methods S1).

### Computational molecular analysis

Secondary structure analysis of transmembrane spanning domain 2 (TSD2) of PfMRP1 was performed with SOSUI server [43]. Computational homology modeling was performed at HHpred-Homology detection & structure prediction by HMM-HMM comparison server (http://toolkit.tuebingen.mpg.de/hhpred). Alignment for best template was performed using Psi-blast with 8 interactions and with local alignment. The template from the best E-score alignment for the second transmembrane spanning domain 2 based in the Msba ABC transporter crystal 3B60, was then used in Modeller software to generate a model. Models were visualized with Yasara software [44].

### Statistical analysis

50% and 90% inhibitory concentrations were calculated by nonlinear regression analysis (http://malaria.farch.net). Statistical analysis was carried out using SigmaPlot for windows version 11.0. Pearson Correlation was used to assess linear relations. *In vitro* drug susceptibility phenotype and genotype association (Table 1 and Table S2) was performed with T-test with equal variances assumed (2-tailed). For comparison, Bonferroni-adjusted level of significance would be: P value <0.0042. Isolates that harbors both alleles (mixed infection) in the determined polymorphic region were excluded from the statistical analysis.

## Supporting Information

Material and Methods S1 PCR program, mastermix and primer sequences for *pfmdr1* and *pfcrt* ORF sequencing and *pfmdr1* F1226Y and *pfmrp1* F1390I genotyping by pyrosequencing.
(DOC)

Table S1 **Comprehensive data of each isolate genotype and** ***in vitro*** **drug susceptibility values.**
(XLS)

Table S2 **Relationship between average in vitro drugs tested IC's and relevant polymorphisms.**
(XLS)
