# Supplementary material for: Novel Polymorphisms in Plasmodium falciparum ABC Transporter Genes Are Associated with Major ACT Antimalarial Drug Resistance
Source: PLoS One. 2011 May 25;6(5):e20212. doi: 10.1371/journal.pone.0020212 (PMC3102103; doi:10.1371/journal.pone.0020212)
Supplement: Material and Methods S1 — PCR program, mastermix and primer sequences for pfmdr1 and pfcrt ORF sequencing and pfmdr1 F1226Y and pfmrp1 F1390I genotyping by pyrosequencing. (DOC) [file pone.0020212.s001.doc]

**Material and Methods S1:**

***pfmdr1* and *pfcrt* ORF sequencing: PCR program, mastermix and primer sequences**

*Pfmdr1* (gDNA) was amplified in 5 fragments and *pfcrt* (cDNA) in 2 fragments in which some had to undergo for 2nd amplification. Amplifications were performed in 50μl with 0.2μM of each forward and reverse primer, 0.2mM dNTPs, 2.5mM MgCl_2_ and 2 Units GoTaq® DNA Polymerase (Promega Biotech AB). The thermal cycle program was 94˚C 5min, and 40 cycles of 94˚C 30sec, annealing temperature 30sec and 72˚C 105sec followed by a longer final extension of 72˚C 7min. Annealing temperature for 1^st^ and 2^nd^ amplification as well as primer sequence for amplification and sequencing are described in the table below.

|  | **Amplified**  **fragment** | **1st amplification primers**  **(5' - 3')** | **Annealing**  **Temp.** | **2nd amplification primers**  **(5' - 3')** | **Annealing**  **Temp.** | **Sequencing primers**  **(5' - 3')** |
| --- | --- | --- | --- | --- | --- | --- |
| ***pfmdr1*** |  |  |  |  |  |  |
|  | 1 | Fw - GTGTACATAGCTTATTTCATTTATA | 53ºC | Fw - GCTTATTTCATTTATAAGATTTA | 53ºC | Fw - GCTTATTTCATTTATAAGATTTA |
|  |  | Rev - ATTGATTCGTTGCACTATTTA |  | Rev - CGAATGCATAAGAAACTAA |  | Rev - CGAATGCATAAGAAACTAA |
|  | 2 | Fw - GCGTTTTTCCGTTAATTTATGT | 53ºC | Fw - CGTTAATTTATGTTTGTGGTGTCAT | 53ºC | Fw - CGTTAATTTATGTTTGTGGTGTCAT |
|  |  | Rev - GTTATTCTATTTTCATTTCCTTTCA |  | Rev - GGATTTCTCATAATTGCTCTTGCA |  | Rev - GGATTTCTCATAATTGCTCTTGCA |
|  | 3 | Fw - GACATCAAATGAATTATTAGAAAT | 51ºC | Fw - CAAACTATTAAAGATTCTGATGTT | 51ºC | Fw - CAAACTATTAAAGATTCTGATGTT |
|  |  | Rev - GCAACAATTGGACAAAAAT |  | Rev - CGTTTCATAGTCTTTTCGACT |  | Rev - CGTTTCATAGTCTTTTCGACT |
|  | 4 | Fw - GCACCAAACAATTTACGTAT | 53ºC |  |  | Fw - GATTATATCCCGTATTTGCT |
|  |  | Rev - CCTGTTTCTCCAACGATT |  |  |  | Rev - GTACATTTGGTCTTGAAATATAA |
|  | 5 | Fw - CTGGTAGTTATGCTGGAAA | 51ºC | Fw - CTTAAAAGGAGATTCAGAAAAT | 51ºC | Fw - GGAGATTCAGAAAATGCAA |
|  |  | Rev - AGGAATATGTCATTAAAATCATAA |  | Rev - CAATATAACGGACAAGAGTTG |  | Rev - CGGACAAGAGTTGATACTGT |
| ***pfcrt*** |  |  |  |  |  |  |
|  | 1 | Fw - CCCAAGTTGTACTGCTTCTAA | 50ºC | Fw - AATAATAAATACACGCAGTCATAT | 44ºC | Fw - AATAATAAATACACGCAGTCATAT |
|  |  | Rev - AATTTTCTTCTTGTGTTTCAA |  | Rev - TTTCTTCTTGTGTTTCAAAAG |  | Rev - TTTCTTCTTGTGTTTCAAAAG |
|  | 2 | Fw - TCAAAAGAACGACACCGAAG | 50ºC |  |  | Fw - TCAAAAGAACGACACCGAA |
|  |  | Rev - ACCTTCAACATTATTCCTTATAA |  |  |  | Fw - CCTGTATGCTTTTCAAACATGAC |
|  |  |  |  |  |  | Rev - ggaaaaaaacatgaaaaataaaa |

***pfmdr1* F1226Y and *pfmrp1* F1390I genotype by pyrosequencing: PCR program, mastermix and primer sequences**

The pyrosequencing protocol and primers were designed with Pyrosequencing assay design software, version 1.0 (Biotage AB, Uppsala, Sweden). *pfmdr1* F1226Y was amplified in a fragment of 183bp and *pfmrp1* F1390I in a fragment of 184bp.

Separate amplifications were performed in 50μl with 0.2μM of each forward and reverse primer (one of them tagged with biotin), 0.2mM dNTPs, 2.5mM MgCl_2_ and 2 Units GoTaq® DNA Polymerase (Promega Biotech AB). The thermal cycle program was 94˚C 3min, and 40 cycles of 94˚C 30sec, 55˚C 30sec and 65˚C 60sec followed by a longer final extension of 65˚C 7min.

After amplification, streptavidin Sepharose beads (Amersham Biosciences, Little Chalfont, United Kingdom) were added to the PCR products, and single-stranded biotinylated PCR products were obtained with a pyrosequencing vacuum prep workstation (Biotage AB, Uppsala, Sweden).

Pyrosequencing reactions with the sequencing primers were performed using the PSQ 96 SNP reagent kit and a PyroMark ID instrument according to the recommendations of the manufacturer (Biotage AB, Uppsala, Sweden). Primer sequence for amplification and sequencing as well as nucleotide dispensation orders are described in the table below.

|  | **Nucleotide**  **change** | **Amplification primers**  **(5’-3’)** | **Amplicon**  **size** | **Sequencing**  **Primer (5’-3’)** | **Nucleotide**  **dispensation order** |
| --- | --- | --- | --- | --- | --- |
| ***pfmdr1* F1226Y** | TTT-TAT | Fw-CAATCTGGATCTGCAGAAGATTA | 183bp | Fw-CTGCAGAAGATTATACTGT | TATATATATATCG |
|  |  | Rev-Biotin-GCATCTTCTCTTCCAAATTTGAT |  |  |  |
| ***pfmrp1* F1390I** | TTT-ATT | Fw-AGCACAAAGGCTGTATTTATCATG | 184bp | Fw-ATTTTCGAATTAGTTATTTT | GATCATGCT |
|  |  | Rev-Biotin-GATGCCCAAATATTTATAAGCCA |  |  |  |
